# Editorial: Cardiovascular and Physical Health in Severe Mental Illness

**DOI:** 10.3389/fpsyt.2021.760250

**Published:** 2021-09-28

**Authors:** Sri Mahavir Agarwal, Anthony Christopher Vernon, Ganesan Venkatasubramanian, Margaret K. Hahn

**Affiliations:** ^1^Schizophrenia Division, Centre for Addiction and Mental Health (CAMH), Toronto, ON, Canada; ^2^Institute of Medical Sciences, University of Toronto, Toronto, ON, Canada; ^3^Department of Psychiatry, University of Toronto, Toronto, ON, Canada; ^4^Banting and Best Diabetes Centre, University of Toronto, Toronto, ON, Canada; ^5^Department of Basic and Clinical Neuroscience, Institute of Psychiatry, Psychology and Neuroscience, King's College London, London, United Kingdom; ^6^Medical Research Council Centre for Neurodevelopmental Disorders, King's College London, London, United Kingdom; ^7^Schizophrenia Clinic, Department of Psychiatry, National Institute of Mental Health and Neurosciences (NIMHANS), Bengaluru, India

**Keywords:** severe mental illness, obesity, cardiovascular health, treatment, prevention

Severe mental illnesses (SMI) such as schizophrenia and bipolar disorder are associated with very high rates of metabolic disorders, including obesity, diabetes, and metabolic syndrome (1–3). There is a marked increase in standardized mortality ratios for both natural and unnatural causes of death in cases of SMI, much of which may be attributed to the increased prevalence of obesity and related coronary heart disease risk factors (4). Beyond cardiovascular risk, metabolic comorbidity has negative implications on other facets of clinical status, including cognitive performance (5–7), stigma (8), social engagement (9), quality of life (10), and adherence with treatment (11). The reasons driving these associations are complex, including contributing effects of illness-associated lifestyle factors, disease biology, and psychotropic treatments. Unfortunately, rates of non-treatment for these medical conditions are high in SMI, representing an unmet medical need (12–16). In this Research Topic, we discuss the relationship between SMI and metabolic dysfunction and provide novel perspectives from neurobiological, pathophysiological, and pharmacological points of view to improve the physical and mental well-being of vulnerable individuals suffering from SMI.

Several included papers have investigated the pathophysiological basis of this relationship. The role of inflammation in the etiopathogenesis of SMI and metabolic dysfunction and the effect of antipsychotics on these processes is an area of active inquiry, and articles in this issue reflect this (17–19). For example, Reponen, Dieset, et al. investigated the correlation between markers of systemic inflammation and dyslipidemia in patients with schizophrenia and bipolar disorder. They report higher levels of inflammatory markers, including high-sensitivity C-reactive protein (CRP) and myeloperoxidase and a correlation between inflammatory markers and atherogenic lipid ratios, suggesting that abnormal neutrophil activation may underlie increased metabolic risk in patients. In a subsequent paper, Reponen, Tesli, et al. have investigated if leptin, adiponectin, or their ratio could predict increased cardiovascular risk in SMI independent of other cardiometabolic risk factors. The group replicates previously documented elevations in leptin in SMI patients (20), but fails to find differences in adiponectin between patients and controls, or according to antipsychotic use. However, adiponectin emerges as a predictor of cardiovascular disease risk across cohorts, suggesting it may be a valuable marker for identifying individuals at higher cardiovascular disease risk. In an elegant preclinical study, Boyda et al. examined the effect of acute exposure to antipsychotics on peripheral catecholamines in female rats and found them to be increased in a manner consistent with their metabolic liability; the smallest increase was seen with haloperidol, followed by risperidone, olanzapine, whilst clozapine exposed rats showed the largest increase. Their findings align with a recent review of the evidence for autonomic dysfunction in schizophrenia where patients on antipsychotics with high-affinity muscarinic antagonism such as clozapine, olanzapine and quetiapine were noted to have the most significant autonomic dysfunction (21).

Delving further into the pathophysiological predictors of cardiovascular risk, Ward et al. combined genetic and metabolomic data to determine the relationship between folate pharmacogenomics, one-carbon metabolites, and insulin resistance. They found that pharmacogenomic variants that decrease the functional capacity of the Methylenetetrahydrofolate reductase (MTHFR) enzyme were associated with increased risk for cardiovascular disease. They also note that the increased presence of methyl-donating group containing molecules such as serine, glycine, and betaine might be inversely correlated with insulin resistance. Kim et al. investigated psychological characteristics associated with food cravings in patients with first-episode psychosis. In a study involving 182 patients (78 males and 104 females), they found that food cravings were, as expected, associated with weight gain. Interestingly, craving was influenced by perceived stress in females. The authors advocate for interventions aimed at reducing perceived stress in female patients with schizophrenia. Sneller et al. have summarized several of these aspects in a systematic review of clinical, biochemical, and genetic factors associated with metabolic syndrome among patients with SMI. They find that higher age, higher baseline BMI, higher current BMI, and male as well as female gender were positively associated with metabolic syndrome across all antipsychotics and suggest that this can form the basis for models that predict the risk of developing metabolic syndrome in patients with SMI who are being treated with antipsychotics.

Kumar et al. investigated the relationship between dietary glutamic acid and depressive symptomatology in patients with schizophrenia, stratified by obesity status, and report a possible correlation between dietary glutamic acid a depressive symptoms. Interestingly, this relationship was not seen in obese patients with schizophrenia supporting existing literature associating obesity, insulin resistance, and inflammation with depressive symptom severity, worse functional outcomes, and brain structural alterations (22). To further follow-up on this line of enquiry, two other studies in this issue addressed effects of metabolic dysfunction on brain structure and function, as measured by magnetic resonance imaging (MRI) across SMIs. Specifically, Kolenič et al. examined whether obesity could explain some of the heterogeneity in brain imaging findings in patients experiencing a first episode of psychosis (FEP). Their findings provide evidence for associations between higher BMI and lower cerebellar volume, raising the possibility that prevention and early mitigation of obesity and its related comorbidity could preserve brain structure in FEP. Taking this idea further, in a hypothesis paper, Calkin et al. have explored the potentially reversible impact of metabolic dysfunction on the brain in bipolar disorder. Leaning on recent findings from their group, they highlight growing evidence for a key role of insulin resistance in bipolar disorder pathophysiology and its relationship to shared inflammatory pathways. They posit that these modulations result in impaired blood-brain barrier (BBB) integrity and worse clinical outcomes and propose that reversing insulin resistance through lifestyle changes, vascular-protective drugs, or insulin-sensitizing medications could be a novel way forward for the prevention or treatment of bipolar disorder.

The special issue also includes two articles that have discussed antipsychotic-induced metabolic dysfunction in special contexts. Libowitz and Nurmi review the impact of this problem in pediatric populations, who represent one of the most vulnerable subgroups for metabolic adverse effects. They go on to propose underlying mechanisms and strategies to mitigate the impact of this large and difficult problem, along with future directions for research. Padmavati et al. have reviewed this problem from the point of view of a developing country, India, and identify strategies that need to be implemented at the clinic, community, and policy levels to understand and address the impact of cardiovascular diseases among persons with schizophrenia in developing countries.

Finally, two papers have discussed potential treatment strategies to address the enormous burden of metabolic dysfunction. García et al. have proposed a protocol to investigate whether implementing an individualized exercise program could improve overall functioning in patients with bipolar disorder. They propose to collect several clinical, biochemical, and imaging-based parameters at baseline, after a 4-month intervention period, and 6-month follow-up to investigate their hypothesis. Kanagasundaram et al. present the results of a large systematic review and meta-analysis assessing the effectiveness of pharmacological interventions in treating dyslipidemia in patients with schizophrenia. They report that antipsychotic switching, antipsychotic add-ons, and certain off label interventions might be effective in improving some but not all associated lipid parameters, highlighting that currently available lipid lowering agents may not work as well in this patient population.

Together, the special issue reviews the large body of work at the intersection of mental and physical health, and includes data from studies conducted across various settings and paradigms including fundamental research, clinical and intervention research, along with new hypothetical frameworks and protocols. It highlights recent advances in the field and that physical and mental well-being are closely interconnected. Concerted efforts to improve physical health are therefore likely to improve both quality of life and overall lifespan among individuals suffering from SMI.

## Author Contributions

SMA and MKH conceptualized the manuscript. SMA authored the first draft. All authors contributed to the writing and editing of the final manuscript.

## Funding

SMA was supported in part by an Academic Scholars Award from the Department of Psychiatry, University of Toronto and the CAMH Discovery Fund, and acknowledges grant support from the Canadian Institutes of Health Research (PJT-426977 and PJT-461675), PSI foundation, Ontario, and the CAMH Discovery Fund. ACV acknowledges financial support from the National Centre for the Replacement, Refinement and Reduction of Animals in Research (NC/S001506/1) and the Medical Research Council (New Investigator Research Grant MR/N025377/1 and Centre Grant MR/N026063/1). GV acknowledges the support of Department of Biotechnology (DBT) - Wellcome Trust India Alliance (IA/CRC/19/1/610005) and Department of Biotechnology, Government of India (BT/HRD-NBA-NWB/38/2019-20(6)). MKH was supported in part by an Academic Scholars Award from the Department of Psychiatry, University of Toronto and has grant support from the Banting and Best Diabetes Center (BBDC), the Canadian Institutes of Health Research (PJT-153262 and PJT-426977), PSI foundation, Ontario, and holds the Kelly and Michael Meighen Chair in Psychosis Prevention, and the Cardy Schizophrenia Research Chair.

## Conflict of Interest

The authors declare that the research was conducted in the absence of any commercial or financial relationships that could be construed as a potential conflict of interest.

## Publisher's Note

All claims expressed in this article are solely those of the authors and do not necessarily represent those of their affiliated organizations, or those of the publisher, the editors and the reviewers. Any product that may be evaluated in this article, or claim that may be made by its manufacturer, is not guaranteed or endorsed by the publisher.
